# Utilizing Developmentally Essential Secreted Peptides Such as Thymosin Beta-4 to Remind the Adult Organs of Their Embryonic State—New Directions in Anti-Aging Regenerative Therapies

**DOI:** 10.3390/cells10061343

**Published:** 2021-05-28

**Authors:** Klaudia Maar, Roland Hetenyi, Szabolcs Maar, Gabor Faskerti, Daniel Hanna, Balint Lippai, Aniko Takatsy, Ildiko Bock-Marquette

**Affiliations:** 1 Department of Biochemistry and Medical Chemistry, Medical School, University of Pecs, Szigeti Street 12., H-7624 Pecs, Hungary; klaudia.maar@aok.pte.hu (K.M.); hetenyiroland@gmail.com (R.H.); maarszabi@gmail.com (S.M.); gaborfaskerti@gmail.com (G.F.); drainor2@gmail.com (D.H.); aniko.takatsy@aok.pte.hu (A.T.); 2 Szentagothai Research Centre, Research Group of Regenerative Science, Sport and Medicine, University of Pecs, Ifjusag Street 20. C301, H-7624 Pecs, Hungary; balint.lippai@gmail.com

**Keywords:** aging, heart regeneration, embryonic development, thymosin beta-4

## Abstract

Our dream of defeating the processes of aging has occupied the curious and has challenged scientists globally for hundreds of years. The history is long, and sadly, the solution is still elusive. Our endeavors to reverse the magnitude of damaging cellular and molecular alterations resulted in only a few, yet significant advancements. Furthermore, as our lifespan increases, physicians are facing more mind-bending questions in their routine practice than ever before. Although the ultimate goal is to successfully treat the body as a whole, steps towards regenerating individual organs are even considered significant. As our initial approach to enhance the endogenous restorative capacity by delivering exogenous progenitor cells appears limited, we propose, utilizing small molecules critical during embryonic development may prove to be a powerful tool to increase regeneration and to reverse the processes associated with aging. In this review, we introduce Thymosin beta-4, a 43aa secreted peptide fulfilling our hopes and capable of numerous regenerative achievements via systemic administration in the heart. Observing the broad capacity of this small, secreted peptide, we believe it is not the only molecule which nature conceals to our benefit. Hence, the discovery and postnatal administration of developmentally relevant agents along with other approaches may result in reversing the aging process.

## 1. Introduction

Aging is an inherent resultant of living, which is well conserved among the species [1]. Based on the United Nation’s database, the percentage of individuals over the age of 65 years is gradually increasing worldwide. Moreover, the median age of the world’s population rose by eight years in the last four decades [2]. Thus, the leading five causes of Disability Adjusted Life Year (DALY) rates are non-communicable age-related diseases [3] placing immense economic, social, and healthcare burdens upon society. Researchers around the globe are conducting a vast number of investigations to better understand the physiological and molecular background of the process of aging. Doubtlessly, increasing longevity proposes the question: how can we prolong our age while assuring quality of life measures in the best physical and mental shape possible.

As we all know, over the span of time, the human body undergoes numerous alterations, including loss of muscle mass, increase in body fat, water loss in the tissues, and decreased bone density, all resulting in varying degenerative processes regarding our physiological make-up [4]. Moreover, the impact of the environment, parental health, lifestyle, and social interactions, much as physical activity during early and later life seemingly bears an equally significant role upon the aging processes and tissue soundness [5,6]. At the cellular and molecular levels, aging is considered to be an accumulation of both internal and external damage, eventually leading to inadequate function of the cell, tissue, and organ. Clearly, the biggest challenge of our era is to understand and successfully intercede with the various molecular pathways responsible for these alterations.

## 2. Hallmarks of Aging

In accordance with our current scientific knowledge, numerous cellular attributes of aging were identified and summarized in various review articles throughout the literature [1,7,8,9,10], all of which are eventually contributing to the decay of the cells and the organism. In addition to identifying the microscopic alterations, there is also an urgent drive to understand the molecular interchanges and to define the molecular markers to depict the pathology behind senescence and recovery. As stated by Niedernhofer and colleagues, while several molecules which were potentially considered for testing and implementation have been successfully identified, the main challenge is to find a unifying mechanism of age-related tissue damage and to prioritize which molecular endpoints to pursue and validate [10]. In following the chronology, investigations first began with initial observations, in which late reproducing organisms live longer than early reproducers. These findings supported that genes may have a significant role in lifespan determination [11]. Today, the actual list of potential influencer genes numbers over 800 [12], jump-starting the identification of numerous well conserved age-related molecular pathways such as insulin-like signaling, mTOR pathway, the discovery of Sirtuins and the importance of NAD^+^ [12]. In the 1950s, research turned towards identifying the role of free radicals and the relevance of the mitochondria in which all results suggested these organelles may be intimately linked to a wide range of processes associated with aging [13]. Animal studies suggested however, that while the levels of mitochondrial mutations increase with age, it remains unclear whether this alteration does truly play a fundamental and significant role in the process [14,15]. In addition to mitochondrial pathology, the presence of various factors which induce permanent inflammation were equally highlighted and detected as potential causes regarding aging. With this, inflammatory senescence (immunosenescence) became a considered marker and hallmark of aging biology [16,17]. Finally, to fully maintain well-functioning healthy cells and protein networks, an extensive machinery of molecular chaperones, proteases, and many other additional factors are equally required [18,19]. In observing all the enlisted factors, one wonders how our morphological, physiological, and molecular knowledge regarding aging will be translated into actual functional therapies? Preventing disease conditions is overly critical in this concept, but reversing the already developed malfunctions is the challenge clinicians face in their daily practice. To support this concept, geroscience, an emerging cross-disciplinary field was established in the last decade with a goal to unravel the molecular and cellular mechanisms responsible for aging as a major risk factor and driver of age-related chronic diseases [20,21,22].

By reviewing the enlisted hallmarks, we observed that most investigations and analyses primarily reconnoiter the journey from the day of our birth, thus leaving the power and knowledge regarding the most dynamic period of our physical development unaccounted and in the dark. To overcome this discrepancy, we suggest the conceptual and therapeutic approach of aging could significantly benefit from observing, extending, and harnessing our knowledge regarding embryonic development, and that utilizing these findings throughout our adult life may significantly re-enhance the regenerative potential of the human body. In the next chapters of this review, we intend to demonstrate and instantiate the viability of this alternative approach in the context of hypoxic heart injury. Naturally, the goal is to employ all future discoveries in the daily routine beside therapy, to increase lifespan and quality of living during old age.

## 3. Pathological Alterations and Recent Therapeutic Approaches to Aid the Adult Heart Following Hypoxia

Many of the adult organs, such as the heart or the brain possess minimal regenerative capacities following tissue damage. In the uninjured heart, approximately 0.5% to 2% of human cardiomyocytes are capable of renewal [23,24,25,26], primarily via proliferation of the existing heart cells or by homing of extracardiac bone marrow-derived cells [26]. Naturally, this rate is far too insufficient to substitute cell loss following cardiac infarction when approximately 25% of the existing cells may be whipped out in a short period of time [25]. Most excitingly, the renewal rate is higher among infants and adolescents than when compared with the elderly, indicating higher regenerative potential among children [23,27]. 

**Figure 1:**
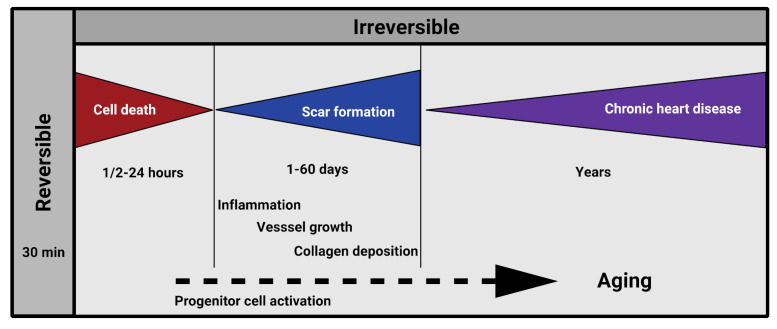
Pathology of post-ischemic cardiac remodeling in humans.

The goal of regenerative therapies is to aid damaged tissues with functionally intact healthy cells of the organ. This may be achieved, for instance, by activating cell proliferation and migration of the viable intact cells, by injection, recruitment, transformation and activation of various progenitor cells, or by combination of bioengineered substances to and at the damaged scene [28,29,30,31,32,33,34,35,36,37,38]. Normally, the damaged organ “solves the problem” by special remodeling of the tissue, which, particularly in the mammalian heart, sadly results in a significant decrease of contractility and function (Figure 1). Recent discoveries suggest that this remodeling process is more a response to hemodynamic or metabolic stress of the heart, which leads to a suppression of the post-natal gene program and results in a predominance of the fetal gene program at the damaged scene [39]. At the level of tissue metabolism, this alteration is reflected by the preference for carbohydrates over fatty acids as substrates regarding energy provision [40]. Naturally, numerous additional factors characteristic for embryonic development (e.g., atrial natriuretic factor (ANF), transforming growth factor β (TGF-β), *c-myc, *c-fos**) as well as isoform switches of many other proteins, including metabolic enzymes and sarcomeric proteins (myosin heavy chains and α-actins) become equally altered [39], which is believed to be more an adaptative response of the adult organ with an aim of stimulating tissue repair and function. 

In consideration of the recent proceedings regarding modern technologies, new generation sequencing (NGS) became a novel tool offering thorough insight into gene alterations occurring during embryonic development and following birth. Strikingly, there are numerous factors which become altered within the first few weeks of life. This was wonderfully summarized by Talman et al. [41], who provide a thorough review regarding mRNA, protein, and metabolite interchanges of the neonatal heart suitable for identification of metabolism-related mechanisms equally associated with cardiac regeneration. Another potential alternative for post-hypoxic cardiac cell replacement may be the induction of adult cardiomyocytes to re-enter the cell cycle and to advance into mitosis and cytokinesis [42] by utilizing pro- and anti-mitotic regulatory RNAs [43,44], long noncoding RNAs [45], proteins, hormones, and other metabolites involved in proliferation pathways [27,46,47]. Naturally, there are many more as yet unknown factors to resuscitate the hypoxic or damaged dying organs. Thus, discovering novel factors critical during development and postnatal alterations via transcriptome analysis may bring substantial regenerative therapies to a close reality [48,49,50,51]. The most limiting factor regarding therapeutical utilization of the described molecules however is that, with small exemptions, they are produced and implement their action primarily in the cytoplasm or in the nucleus of the cells. Thus, systemic administration for most of the factors as a potential therapeutic agent may be restricted. 

As mentioned, an additional alternative for creating a clinical imperative to offset cardiac cell death during acute ischemic injury and to promote cardiac function is the utilization of intra- or extracardiac stem cells [24,28,29,30,33,34,35,37,52,53,54,55,56,57]. Although the stem cell population could maintain a balance between cell death and renewal under normal circumstances, despite its promises regarding heart failure therapy [38], it is sadly insufficient for proper repair following infarction. Thus, introduction of extracardiac progenitors such as mesenchymal or embryonic endothelial precursors may better support functionality. This approach, however, has been deemed controversial [56,58] as the technical hurdles regarding progenitor cell delivery and differentiation have thus far prevented broad clinical applications following hypoxia [28].

It is well accepted that brief hypoxic periods in the heart activate cardioprotective mechanisms and enhance cardiomyocyte tolerance to ischemia (ischemic preconditioning (IP)) [59,60,61,62]. A humoral mechanism of cardioprotection by remote ischemic preconditioning (RIP) has been equally demonstrated in various models of ischemia–reperfusion including upper and lower extremities, liver, and the mesenteric and renal arteries [62]. Due to the influence of the RIP induced secreted molecules, signal transduction mechanisms are triggered resulting in protection of the various organs from later injuries. Surprisingly, protection can be transferred even across species with plasma-derived dialysate containing nitric oxide, stromal derived factor-1α, microRNA-144, and many not yet identified components. The initiated intracardiac signal transduction involves adenosine, bradykinin, various cytokines, and chemokines activating specific receptors, kinases, or mitochondrial function [60].

Considering the positive impact of hypoxia initiated secreted factors and due to clinical obstacles with stem cell therapies, it is tempting to find and utilize various small molecules such as peptides, micropeptides or miRNAs, secreted by progenitor cells or other cell types to support cardiac cell survival, vessel growth, and function [30,63,64]. In the following parts of this review, we introduce a developmentally critical, postnatally altered and naturally existing small, secreted molecule capable of fulfilling the enlisted criteria with a potential of becoming a therapeutic candidate for repairing the damaged tissue of various organs in humans.

## 4. Beta Thymosins

**Figure 2:**
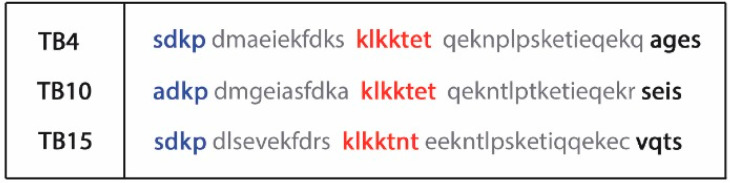
Amino acid sequence and domain structure of human beta thymosins. Actin binding domain (red); N-terminal variable domains (blue), and C-terminal variable domains (black) are interconnected by two alpha helixes (gray). (TB4: Thymosin beta-4, TB10: Thymosin beta-10, TB15: Thymosin beta-15. (Source: https://www.ncbi.nlm.nih.gov/Protein; accessed on 20 May 2021)).

The beta thymosins are a family of highly conserved acidic 5 kDa peptides, originally thought to be thymic hormones and first described in 1966 by A.L. Goldstein and A. White [65]. Currently, sixteen beta thymosins are known, of which, only three are present in humans: Thymosin β4 (TB4), Thymosin β10 (TB10), and Thymosin β15 (TB15) (Figure 2). 

While they possess a highly conserved and homologous structure (43–44 amino-acid residues), they are expressed from from variant gene products, and thus they are biochemically and functionally distinct molecules [66]. Beta thymosins differ from alpha thymosins upon their isoelectric point: below 5 are termed α and between 5 and 7 are termed β [67]. TB4 is the main peptide, representing nearly 70–80% of the total beta thymosin content [68].

Structurally, TB4 contains a central actin-binding domain surrounded by two alpha helices and an amino (N) and carboxy (C) terminal variable region (Figure 2). It is present in high concentrations (up to 0.4 mM) in various adult tissues, especially in the spleen, lungs, thymus, brain, and heart [69], as well as in macrophages, tumor and human blood cells, while serum contains less than 1% of the TB4 amount present in the entire blood system. Apart from blood, TB4 may be equally found in wound fluid or in additional body fluids, such as saliva or tears [67,70,71,72].

## 5. The Expression of Thymosin Beta-4 during Embryonic Development

**Figure 3:**
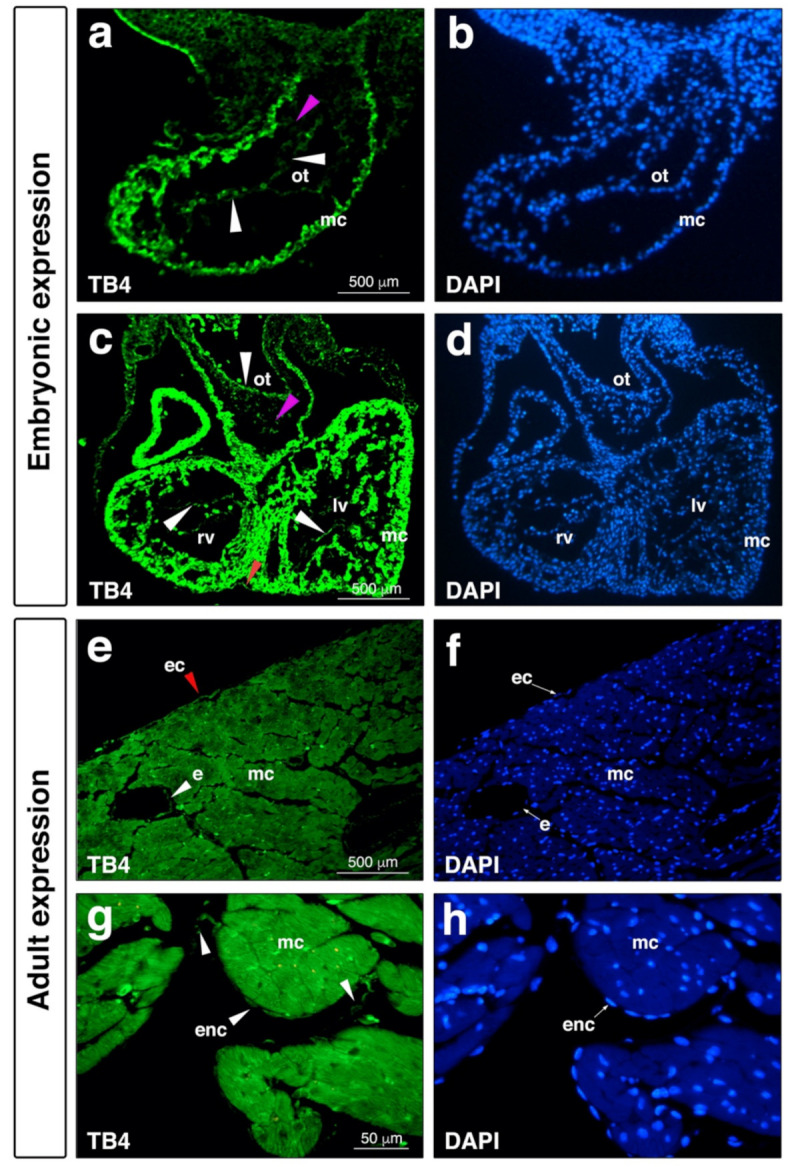
Thymosin β4 is expressed in endocardial, coronary endothelial, and epicardial cells during embryonic development and in adult mice. (**a–d**), Positive thymosin β4 expression (light green) (**a**,**c**) and DAPI staining (blue) (**b**,**d**) in E9.5 mouse embryonic endocardial cells (**a**, white arrowhead), and in E11.5 embryonic endocardial (**c**, white arrowhead) and epicardial cells (**c**, red arrowhead). Note absence or very low levels of thymosin β4 expression in mesenchymal cells (**a**,**c**, magenta arrowhead). (**e**–**h**), Immunostaining of adult mouse hearts reveals positive thymosin β4 expression in the epicardium (**e**, red arrowhead), vessel endothelium (**e**, white arrowhead) and endocardium (**g**, white arrowhead). (**f**,**h**) are DAPI staining of (**e**,**g**). ot, outflow tract; rv, right ventricle; lv, left ventricle; mc, myocardium; ec, epicardium; e, vessel endothelium; enc, endocardium.

As suggested, investigation of a molecule’s pre- and postnatal expression may presumably provide significant information regarding its potential clinical utilization and appliance. According to the present and earlier literature, beta thymosins are widely expressed among various tissues such as the brain, kidneys, heart, skin, eyes and cell types of the body [73,74,75,76,77,78,79,80,81,82,83,84]. The first investigations were communicated by Lin and colleagues in 1990, when they presented significant mRNA expression of both TB10 and TB4 in the cortex and cerebellum [73]. Since then, numerous results have been published suggesting the ubiety of beta thymosins among various species including fish, chickens, and humans [73,74,75,80,85]. TB4′s presence in the heart was first described by Gomez-Marquez thirty years following the molecule’s discovery [86]. The authors observed a hybridization signal in several areas of the embryo, especially in blood vessels and in heart tissue, suggesting a role for the peptide in angiogenesis [86]. It was in 2004, when the next significant observations were made regarding TB4′s presence during mouse heart development [87]. As demonstrated, the molecule is expressed in the left ventricle, outer curvature of the right ventricle, and cardiac outflow tract of the developing heart. Radioactive in situ hybridization indicated TB4 transcripts were enriched in the region of cardiac valve precursors known as endocardial cushions. Cells in this region are derived from endothelial cells which undergo mesenchymal transformation and invade a swelling of extracellular matrix separating the myocardium and endocardium. TB4-expressing cells in the cushions co-expressed muscle actin, suggesting TB4 was present in migratory cardiomyocytes known to invade the endocardial cushion [88]. In the ventricles, TB4 transcripts were found at E9.5–E12.5 in the septum and the compact layer, the most proliferative region of the myocardium. The outflow tract myocardium which migrates from a secondary heart field and the developing epicardium also expressed high levels of TB4 mRNA [79,89]. The supportive immunohistochemical analyses fortified mRNA expression (Figure 3). Finally, in addition to the expression in the heart, whole mount mRNA analyses revealed the peptide is equally expressed in the brain, pharyngeal arches, eyes, limb buds, and myotomes, suggesting a potential role for the molecule during the development of these organs in mice [87].

Excitingly, recent analyses of TB4 in human embryos confirmed the presence of the peptide in the developing human heart [81]. In contrast to our findings demonstrating that TB4 co-localizes clearly with muscle actin positive cells in murine embryos, Saunders et al. did not find TB4 in the ventricular myocardium. In human embryos, TB4 protein was primarily localized in endothelial cells of the heart, including the medium coronary vessels. The expression of TB4 in the atria, epicardium, and endocardium, however, was equally confirmed in mice and humans [81]. 

To better understand the function of TB4 during embryonic development, knockout and knockdown models were both created. However, due to differing techniques utilized for knockout production, the results of these studies became controversial. The discrepancies between the results of the straight knockout studies and those of shRNA knockdown studies likely resulted from the distinct experimental approaches used for inhibiting/blocking TB4 [79,90,91,92]. Nonetheless, the studies suggested a significant impact of TB4 on vessel growth [91,93]. Finally, by investigating the impact of TB4 during gestation, most recent reports suggest administration of the peptide may act as a powerful growth promoter by accelerating the development of newborn organs and tissues in mice [94]. 

## 6. Thymosin Beta-4 Holds Significant Potential to Initiate Organ Regeneration and Repair by Influencing Multiple Senescence Related Biological Platforms in Adult Mammals

Identification of the hallmarks of senescence defined numerous potential target points in reversing or influencing age-related degenerative processes [1,7,8,10,16,17,95,96]. To translate these findings regarding the hypoxic heart, it is equally critical to precisely define the targets of the human infarction pathology to successfully inhibit or reverse functional loss. These factors include inhibition of cardiac cell death, inflammation, and scar formation (Figure 1) [97]. 

In focusing on TB4′s influence upon inflammatory processes, earlier data revealed monocytes produce TB4 sulfoxide in response to glucocorticoid administration. TB4 sulfoxide was discovered to block neutrophil chemotaxis in vitro and to possess a potent anti-inflammatory activity in vivo [98]. Moreover, immunomodulatory effects of TB4 prevented apoptosis by decreasing cytochrome c release from mitochondria by increasing BCL-2 expression and decreasing caspase activation [99]. We know TB4 also plays a role during sepsis, a dysregulated host response to infection resulting in life-threatening organ damage. A sustained F-actinemia may create endothelial injury and microthrombi characteristic of sepsis pathology. While F-actinemia is present in sepsis, TB4, as an actin-binding protein, inhibited polymerization of F-actin. Currently, ongoing studies utilize the peptide’s regenerative power in the infected or injured eye (Pseudomonas aeruginosa-induced keratitis [100], corneal wound healing [101]) or, as a doping agent, to enhance performance and skeletal muscle regeneration (TB500) [102]. In dermal phase II trials, TB4 promoted wound healing by accelerating the rate of repair. Patients with pressure ulcers, stasis ulcers, and epidermolysis bullosa benefited from TB4 therapy. The results concluded TB4 is safe, well-tolerated and possessed additional use for skin regeneration [103,104].

The significance of TB4 regarding cardiac regeneration and repair was first indicated in a publication by members of our research group, affiliating a leading role for the peptide in aiding cardiac function following hypoxia [87]. We discovered that external administration of TB4 promotes myocardial cell migration and survival in embryonic tissue in vitro and retains this property following birth. After coronary artery ligation in mice, the peptide enhanced myocyte survival and improved cardiac function, suggesting TB4 may be a novel therapeutic target in the setting of acute myocardial damage from heart attacks and other myocardial diseases among children and adults. Remarkably, the degree of improvement when TB4 was administered systemically through intraperitoneal injections or only locally within the cardiac infarct was not statistically different, suggesting the beneficial effects of TB4 likely occurred through a direct effect on cardiac cells rather than through an extracardiac source [87]. While investigating the potential mechanisms and interactors through which TB4 may be influencing myocardial cell migration and survival events via phage display, we proved ILK-mediated Akt activation to inhibit myocadial cell death may be one of the role players in the process. The observations in vivo were consistent with the effects of TB4 on cell migration and survival demonstrated in vitro and suggest activation of ILK and subsequent stimulation of Akt may, in part, explain the enhanced cardiomyocyte survival induced by TB4, although it was clearly unlikely that only this single mechanism is responsible for the full repertoire of TB4′s cellular effects [87]. Moreover, multimorbid analyses of diabetic and dyslipidemic porcine ischemic heart models equally revealed an attractive therapeutic potential of adeno-associated virus encoding TB4 in ischemic heart disease [105], supporting the described benevolent effects of TB4′s administration following cardiac injury. Since the original discoveries, numerous supporting reports have been published regarding the positive impact of TB4 or its N- or C-terminal domains on cardiac regeneration and repair, suggesting the molecule has a real potential to positively influence age-initiated alterations in the adult human heart and vessels [93,106,107,108,109,110,111,112,113,114,115]. 

## 7. Activating the Embryonic Developmental Program May Be the Key for Thymosin Beta-4′s Regenerative Potential in the Heart

**Figure 4:**
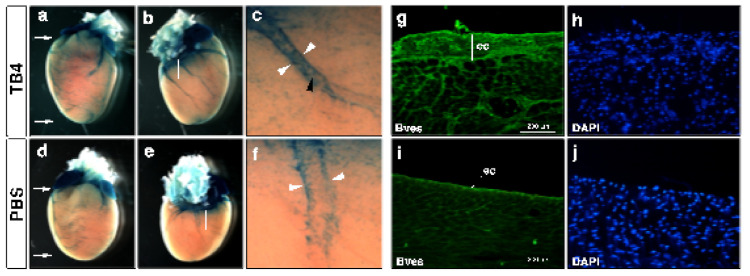
Thymosin β4 activates capsulin positive progenitors in uninjured hearts and initiates organ-wide epicardial thickening at the non-infarcted areas of the infarcted adult mice heart in vivo. (**a**–**f**), Thymosin β4 administration (**a**–**c**) activates perivascular (c white arrowheads) and vascular (c black arrowhead) capsulin positive cells when compared to PBS controls (**d**–**f**). (**a**,**d**) are frontal, (**b**,**e**) are dorsal views of representative thymosin β4 or PBS treated hearts. (**c**,**f**) are high power images of the boxed areas in (**b**,**e**). (**g**–**j**), Immunohistochemical analysis with anti-Bves antibody shows significant increase in Bves-positive cells of epicardial origin and organ-wide thickening of the epicardium along with a significant increase of the number of capillaries and mature vessels 3 days after systemic thymosin β4 treatment (**g**) compared to PBS (**i**). (**h**,**j**) are DAPI stain of (**g**,**i**). ec: epicardium.

As we know from earlier studies regarding zebrafish, the adult fish heart is capable of complete regeneration following a severe injury [116]. The primary reason behind this incredible regenerative potential was associated to a single cell layer blanketing the adult heart, referred to as the epicardium [116]. Morphologically, the epicardium is an active multiple cell layer in the embryo and contains various progenitor cells which provide a source for vascular and even myocardial cell population of the heart [117,118,119,120]. Among adults, this multilayer character becomes single, and the cell layer inactive and dormant, with the exception of tissue injury (Figure 4i) [121]. In the past ten years, numerous studies have been launched to reveal and understand the regenerative potential of this unique mesothelial cell population. As we know, in the embryo, epicardial cells derive from a transient cell cluster called the proepicardial organ (PEO) [122]. During development, these cells translocate to the myocardial surface, where a subset of the cell population undergoes an epithelial-to-mesenchymal transition (EMT), moving into the subepicardial space. These so-called epicardium derived cells (EPDCs) then give rise to various cell types of the heart [123]. For instance, in mice they are the major source for cardiac fibroblasts, vascular smooth muscle cells and vessel pericytes [124,125], cardiomyocytes [126], and cardiac adipocytes [127]. Following birth, hypoxia can re-activate the epicardial layer and the embryonic gene program in the close proximity to the injury, thus providing support for regular adult tissue repair and scar formation [118]. Since the epicardium has such an impressive regenerative potential, as witnessed in adult fish or injured neonatal mice [128], we asked whether this activation can be equally achieved in the adult mammalian heart. To our astonishment, intravenous injections of TB4 did alter the morphology of the adult epicardium, and the changes resembled the characteristics of the embryo. Namely, TB4 administration resulted in the thickening of the epicardial monolayer not only at the infarcted site but also at the remote, healthy areas of the heart [115]. In addition to morphology, a re-activation of the embryonic program was equally reflected by the increased number of capillaries and mature cardiac vessels and by the alteration of the gene expression profile typical of the embryonic state (Figure 4g–j) [115]. Supporting these findings, recent results utilizing a TB4 releasing functional self-assembling peptide also demonstrated the molecule promotes proliferation, migration, and differentiation of EPDCs into cardiovascular and lymphatic cell lineages of the adult hypoxic mouse heart in vivo [113]. Observing the effects of TB4 on post-hypoxic epicardial activation, the question remains as to whether injury is a requirement for the activation process or does the molecule itself initiate the regenerative program and, in doing so, reset time and thus potentially inhibit senescence? To answer this question, we analyzed the expression of Capsulin (pod-1/epicardin), a bHLH transcription factor formerly described to be expressed in mesenchymal cells at sites of epithelial–mesenchymal interactions in developing cardiovascular systems and in the epicardium [129]. To investigate the effect of TB4 on Capsulin positive cells in vivo, we treated heterozygous capsulin/LacZ animals systemically with TB4 or with PBS for six days without cardiac ligation. We found TB4 visibly increased the number of capsulin positive progenitor cells in the coronaries, atrioventricular valves, and epicardium when compared with controls, indicating that TB4 is not only capable of capsulin positive epicardial progenitor activation in vivo but also that the effect is independent of hypoxic injury (Figure 4a–f). Our results clearly demonstrate that TB4 is capable of re-activating embryonic processes, thus, rewinding the biological clock in the adult heart simply by systemic administration. 

## 8. Future Perspectives

**Figure 5:**
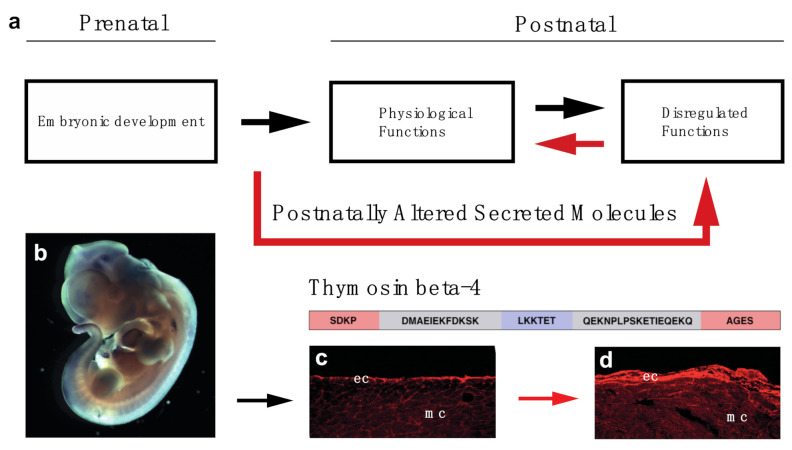
Concept of utilizing prenatally active secreted molecules to restore organ function of the elderly (**a**). Thymosin beta-4 is expressed during embryonic development and can re-activate the epicardium of the adult mouse heart: (**b**), Whole mount in-situ hybridization utilizing Thymosin beta-4 mRNA in E12.5 mouse embryo. c, d: Representative immunohistochemical images utilizing anti-Bves antibody (red) 3 days after PBS (**c**) and Thymosin beta-4 (**d**) treatment indicate systemic injection of Thymosin beta-4 is capable of transforming the adult epicardium to an embryonically active state at the uninjured, healthy areas of the heart. Schematic represents the amino acid and domain structure of Thymosin beta-4. (red: N- and C-terminal variable domains; blue: G-actin binding domain; gray: alpha helixes) ec: epicardium, mc: myocardium.

Reversing age-related alterations of the body is a long-desired goal of humankind. To achieve this goal, it is critical to first precisely understand, analyze and define the macroscopic, cellular and molecular processes of age-related alterations of the body. In this review, our focus highlights aspects of the heart, in which we outlined some of the alternatives scientists utilize to avoid cellular death and to restore, as yet without much success, the physiological conditions. Due to the complexity of the picture, however, reversing the negative processes of organ damage or aging is nearly impossible. To overcome complications and technical hurdles, we propose instead of stopping or fixing aging related processes, we redirect the adult organ towards its earlier developmental state with the hope this approach may also overcome the significant impact of environmental influence and genetic variations, since early development of the human organo- and embryogenesis seems to be more universal (Figure 5). 

Despite the extravagant premise, the dream of returning to our youth may not be so far-fetched. Nature actually provides an enormous list of molecules, some of which are silenced after birth but could serve as a potential treatment to reverse age, at least at the level of a single yet complicated organ such as the heart. The question remains: can postnatal increase of developmentally relevant proteins and peptides reverse organ ageing or damage in a beneficial way? We believe the answer to this question is yes. In our earlier research, we introduced a naturally secreted small molecule, Thymosin beta-4, which is capable of such miracles not only regarding the heart but in the brain and kidneys [69,130]. The protein was first purified from the thymus and binds and alters the cytoskeletal actin filaments by sequestering actin monomers and in doing so, influences actin filament assembly and regulates migration of various cell types such as endothelial cells, myocardial cells or epicardial progenitors [115]. It equally inhibits cellular death by activating and binding numerous role players of the focal adhesion complex, which, eventually, results in the activation of Akt, a proven substrate of ILK with wide-ranging signalling functions that affect growth, survival and motility [87]. Naturally, all the other mechanisms by which TB4 initiates the increase of cardiac function are still under investigation by a host of scientists worldwide. Undoubtedly, its capability in activating the adult epicardium and its ability to resemble its embryonic function without risking injury suggests grounds for hope that the molecule does achieve the same in other organs.

Reminding adult cells of their highly proliferative state is not without risk, as the applied treatment may easily result in unwanted malignancies. Although TB4 was reported to be a prognostic marker for highly metastatic cancer states and its tumor promo-ting properties were equally demonstrated [131,132], its true nature regarding tumorigenesis is controversial. In our hands, the molecule significantly inhibited the progression of pancreatic cancer following systemic administration in mice in vivo (unpublished results). An additional factor supporting this result is that TB4 was equally introduced as a candidate tumor suppressor in male breast cancer [133] and was demonstrated to have a tumor suppressive function in myeloma development by other research teams [134,135]. In addition, the high safety profile observed in Phase I and Phase II clinical trials this far strongly anticipate that TB4 will be safe and efficacious at the applied local or systemic dose for broad clinical applications in the future [136].

We still do not know all the details regarding this special molecule; however, we genuinely believe there are more like it concealed in the human body, awaiting discovery. With their help, we may be capable of fulfilling our dream of reversing age, and as brilliantly foreseen by Professor Schneider [137], perhaps we will be able to create an alternative for the legend, and Prometheus will soon be unleashed and no longer bound.

## Data Availability

No new data were created or analyzed in this study. Data sharing is not applicable to this article.

## References

[1] López-Otín C., Blasco M.A., Partridge L., Serrano M., Kroemer G. (2013). The Hallmarks of Aging. Cell.

[2] United Nations (2019). World Population Prospects 2019.

[3] GBD 2019 Diseases and Injuries Collaborators (2020). Global burden of 369 diseases and injuries in 204 countries and territories, 1990–2019: A systematic analysis for the Global Burden of Disease Study 2019. Lancet.

[4] JafariNasabian P., Inglis J., Reilly W., Kelly O.J., Ilich J.Z. (2017). Aging human body: Changes in bone, muscle and body fat with consequent changes in nutrient intake. J. Endocrinol..

[5] Bouaziz W., Lang P.O., Schmitt E., Kaltenbach G., Geny B., Vogel T. (2016). Health benefits of multicomponent training programmes in seniors: A systematic review. Int. J. Clin. Pract..

[6] Jenkin C.R., Eime R.M., Westerbeek H., O’Sullivan G., Van Uffelen J.G.Z. (2017). Sport and ageing: A systematic review of the determinants and trends of participation in sport for older adults. BMC Public Health.

[7] Khan S.S., Singer B.D., Vaughan D.E. (2017). Molecular and physiological manifestations and measurement of aging in humans. Aging Cell.

[8] Hernandez-Segura A., Nehme J., Demaria M. (2018). Hallmarks of Cellular Senescence. Trends Cell Biol..

[9] Partridge L., Deelen J., Slagboom P.E. (2018). Facing up to the global challenges of ageing. Nat. Cell Biol..

[10] Niedernhofer L., Kirkland J., Ladiges W. (2017). Molecular pathology endpoints useful for aging studies. Ageing Res. Rev..

[11] Rose M.R., Charlesworth B. (1980). A test of evolutionary theories of senescence. Nat. Cell Biol..

[12] Campisi J., Kapahi P., Lithgow G.J., Melov S., Newman J.C., Verdin E. (2019). From discoveries in ageing research to therapeutics for healthy ageing. Nat. Cell Biol..

[13] Sun N., Youle R.J., Finkel T. (2016). The Mitochondrial Basis of Aging. Mol. Cell.

[14] Trifunovic A., Wredenberg A., Falkenberg M., Spelbrink J.N., Rovio A.T., Bruder C.E., Bohlooly-Y M., Gidlöf S., Oldfors A., Wibom R. (2004). Premature ageing in mice expressing defective mitochondrial DNA polymerase. Nat. Cell Biol..

[15] Katajisto P., Döhla J., Chaffer C.L., Pentinmikko N., Marjanovic N., Iqbal S., Zoncu R., Chen W.W., Weinberg R.A., Sabatini D.M. (2015). Asymmetric apportioning of aged mitochondria between daughter cells is required for stemness. Science.

[16] Rodrigues L.P., Teixeira V.R., Alencar-Silva T., Simonassi-Paiva B., Pereira R.W., Pogue R., Carvalho J.L. (2021). Hallmarks of aging and immunosenescence: Connecting the dots. Cytokine Growth Factor Rev..

[17] Zhou D., Borsa M., Simon A.K. (2021). Hallmarks and detection techniques of cellular senescence and cellular ageing in immune cells. Aging Cell.

[18] Jayaraj G.G., Hipp M.S., Hartl F.U. (2020). Functional Modules of the Proteostasis Network. Cold Spring Harb. Perspect. Biol..

[19] Hipp M.S., Kasturi P., Hartl F.U. (2019). The proteostasis network and its decline in ageing. Nat. Rev. Mol. Cell Biol..

[20] Burch J.B., Augustine A.D., Frieden L.A., Hadley E., Howcroft T.K., Johnson R., Khalsa P.S., Kohanski R.A., Li X.L., Macchiarini F. (2014). Advances in Geroscience: Impact on Healthspan and Chronic Disease. J. Gerontol. Ser. A.

[21] Sierra F. (2016). Moving Geroscience Into Uncharted Waters. J. Gerontol. Ser. A.

[22] Fulop T., Larbi A., Khalil A., Cohen A.A., Witkowski J.M. (2019). Are We Ill Because We Age?. Front. Physiol..

[23] Bergmann O., Bhardwaj R.D., Bernard S., Zdunek S., Barnabé-Heider F., Walsh S., Zupicich J., Alkass K., Buchholz B.A., Druid H. (2009). Evidence for Cardiomyocyte Renewal in Humans. Science.

[24] Senyo S.E., Steinhauser M.L., Pizzimenti C.L., Yang V.K., Cai L., Wang M., Wu T.-D., Guerquin-Kern J.-L., Lechene C.P., Lee R.T. (2013). Mammalian heart renewal by pre-existing cardiomyocytes. Nat. Cell Biol..

[25] Laflamme M.A., Murry C.E. (2011). Heart regeneration. Nat. Cell Biol..

[26] Eschenhagen T., Bolli R., Braun T., Field L.J., Fleischmann B.K., Frisen J., Giacca M., Hare J.M., Houser S., Lee R.T. (2017). Cardiomyocyte Regeneration: A Consensus Statement. Circulation.

[27] Mollova M., Bersell K., Walsh S., Savla J., Das L.T., Park S.-Y., Silberstein L.E., Dos Remedios C.G., Graham D., Colan S. (2013). Cardiomyocyte proliferation contributes to heart growth in young humans. Proc. Natl. Acad. Sci. USA.

[28] Zhu D., Cheng K. (2021). Cardiac Cell Therapy for Heart Repair: Should the Cells Be Left Out?. Cells.

[29] Worku M.G. (2021). Pluripotent and Multipotent Stem Cells and Current Therapeutic Applications: Review. Stem Cells Cloning.

[30] Witman N., Zhou C., Beverborg N.G., Sahara M., Chien K.R. (2020). Cardiac progenitors and paracrine mediators in cardiogenesis and heart regeneration. Semin. Cell Dev. Biol..

[31] Sebastião M.J., Marcos-Silva L., Gomes-Alves P., Alves P.M. (2021). Proteomic and Glyco(proteo)mic tools in the profiling of cardiac progenitors and pluripotent stem cell derived cardiomyocytes: Accelerating translation into therapy. Biotechnol. Adv..

[32] Riching A.S., Song K. (2021). Cardiac Regeneration: New Insights into the Frontier of Ischemic Heart Failure Therapy. Front. Bioeng. Biotechnol..

[33] James E., Tomaskovic-Crook E., Crook J. (2021). Bioengineering Clinically Relevant Cardiomyocytes and Cardiac Tissues from Pluripotent Stem Cells. Int. J. Mol. Sci..

[34] Huang J., Feng Q., Wang L., Zhou B. (2021). Human Pluripotent Stem Cell-Derived Cardiac Cells: Application in Disease Modeling, Cell Therapy, and Drug Discovery. Front. Cell Dev. Biol..

[35] Hsia G.S.P., Esposito J., da Rocha L.A., Ramos S.L.G., Okamoto O.K. (2021). Clinical Application of Human Induced Pluripotent Stem Cell-Derived Organoids as an Alternative to Organ Transplantation. Stem Cells Int..

[36] Hashemzadeh M.R., Yazdi M.E.T., Amiri M.S., Mousavi S.H. (2021). Stem cell therapy in the heart: Biomaterials as a key route. Tissue Cell.

[37] Campostrini G., Windt L.M., van Meer B.J., Bellin M., Mummery C.L. (2021). Cardiac Tissues from Stem Cells: New Routes to Maturation and Cardiac Regeneration. Circ. Res..

[38] Bolli R., Solankhi M., Tang X.-L., Kahlon A. (2021). Cell Therapy in Patients with Heart Failure: A Comprehensive Review and Emerging Concepts. Cardiovasc. Res..

[39] Rajabi M., Kassiotis C., Razeghi P., Taegtmeyer H. (2007). Return to the fetal gene program protects the stressed heart: A strong hypothesis. Heart Fail. Rev..

[40] Taegtmeyer H., Sen S., Vela D. (2010). Return to the fetal gene program: A suggested metabolic link to gene expression in the heart. Ann. N.Y. Acad. Sci..

[41] Talman V., Teppo J., Pöhö P., Movahedi P., Vaikkinen A., Karhu S.T., Trošt K., Suvitaival T., Heikkonen J., Pahikkala T. (2018). Molecular Atlas of Postnatal Mouse Heart Development. J. Am. Heart Assoc..

[42] Locatelli P., Belaich M.N., Lopez A., Olea F.D., Urnaga Vega M., Gimenez C.S., Simonin J.A., Bauza M.D., Castillo M.G., Cuniberti L.A. (2020). Novel insights into cardiac regeneration based on differential fetal and adult ovine heart transcriptomic analysis. Am. J. Physiol. Circ. Physiol..

[43] Katz M.G., Fargnoli A.S., Kendle A.P., Hajjar R.J., Bridges C.R. (2016). The role of microRNAs in cardiac development and regenerative capacity. Am. J. Physiol. Circ. Physiol..

[44] Wu Y.H., Zhao H., Zhou L.P., Zhao C.X., Wu Y.F., Zhen L.X., Li J., Ge D.X., Xu L., Lin L. (2015). miR-134 Modulates the Proliferation of Human Cardiomyocyte Progenitor Cells by Targeting Meis2. Int. J. Mol. Sci..

[45] Ponnusamy M., Liu F., Zhang Y.-H., Li R.-B., Zhai M., Liu F., Zhou L.-Y., Liu C.-Y., Yan K.-W., Dong Y.-H. (2019). Long Noncoding RNA CPR (Cardiomyocyte Proliferation Regulator) Regulates Cardiomyocyte Proliferation and Cardiac Repair. Circulation.

[46] Porrello E.R., Mahmoud A.I., Simpson E., Hill J.A., Richardson J.A., Olson E.N., Sadek H. (2011). Transient Regenerative Potential of the Neonatal Mouse Heart. Science.

[47] Porrello E.R., Mahmoud A.I., Simpson E., Johnson B.A., Grinsfelder D., Canseco D., Mammen P.P., Rothermel B.A., Olson E.N., Sadek H.A. (2013). Regulation of neonatal and adult mammalian heart regeneration by the miR-15 family. Proc. Natl. Acad. Sci. USA.

[48] Quaife-Ryan G.A., Sim C.B., Ziemann M., Kaspi A., Rafehi H., Ramialison M., El-Osta A., Hudson J.E., Porrello E.R. (2017). Multicellular Transcriptional Analysis of Mammalian Heart Regeneration. Circulation.

[49] Hu P., Liu J., Zhao J., Wilkins B.J., Lupino K., Wu H., Pei L. (2018). Single-nucleus transcriptomic survey of cell diversity and functional maturation in postnatal mammalian hearts. Genes Dev..

[50] Gan J., Sonntag H.-J., Tang M.K., Cai D., Lee K. (2015). Integrative Analysis of the Developing Postnatal Mouse Heart Transcriptome. PLoS ONE.

[51] De Laughter D.M., Bick A.G., Wakimoto H., McKean D., Gorham J.M., Kathiriya I.S., Hinson J.T., Homsy J., Gray J., Pu W. (2016). Single-Cell Resolution of Temporal Gene Expression during Heart Development. Dev. Cell.

[52] Anversa P., Nadal-Ginard B. (2002). Myocyte renewal and ventricular remodelling. Nat. Cell Biol..

[53] Alvarado-Ramy F., Beltrami E.M. (2003). New guidelines for occupational exposure to blood-borne viruses. Clevel. Clin. J. Med..

[54] Beltrami A.P., Barlucchi L., Torella D., Baker M., Limana F., Chimenti S., Kasahara H., Rota M., Musso E., Urbanek K. (2003). Adult Cardiac Stem Cells Are Multipotent and Support Myocardial Regeneration. Cell.

[55] Orlic D., Kajstura J., Chimenti S., Jakoniuk I., Anderson S.M., Li B., Pickel J., McKay R.D.G., Nadal-Ginard B., Bodine D.M. (2001). Bone marrow cells regenerate infarcted myocardium. Nat. Cell Biol..

[56] Murry C.E., Soonpaa M.H., Reinecke H., Nakajima H., Nakajima H.O., Rubart M., Pasumarthi K.B.S., Virag J.I., Bartelmez S.H., Poppa V. (2004). Haematopoietic stem cells do not transdifferentiate into cardiac myocytes in myocardial infarcts. Nat. Cell Biol..

[57] Ieda M., Fu J.-D., Delgado-Olguin P., Vedantham V., Hayashi Y., Bruneau B.G., Srivastava D. (2010). Direct Reprogramming of Fibroblasts into Functional Cardiomyocytes by Defined Factors. Cell.

[58] Balsam L.B., Wagers A.J., Christensen J.L., Kofidis T., Weissman I.L., Robbins R.C. (2004). Haematopoietic stem cells adopt mature haematopoietic fates in ischaemic myocardium. Nat. Cell Biol..

[59] Heusch G. (2015). Molecular basis of cardioprotection: Signal transduction in ischemic pre-, post-, and remote conditioning. Circ. Res..

[60] Heusch G., Bøtker H.E., Przyklenk K., Redington A., Yellon D. (2015). Remote Ischemic Conditioning. J. Am. Coll. Cardiol..

[61] Sprick J.D., Mallet R.T., Przyklenk K., Rickards C.A. (2019). Ischaemic and hypoxic conditioning: Potential for protection of vital organs. Exp. Physiol..

[62] Tsibulnikov S.Y., Maslov L.N., Gorbunov A.S., Voronkov N.S., Boshchenko A.A., Popov S.V., Prokudina E.S., Singh N., Downey J.M. (2019). A Review of Humoral Factors in Remote Preconditioning of the Heart. J. Cardiovasc. Pharmacol. Ther..

[63] Shrivastava S., Srivastava D., Olson E.N., DiMaio J.M., Bock-Marquette I. (2010). Thymosin β4 and cardiac repair. Ann. N.Y. Acad. Sci..

[64] Magadum A., Kaur K., Zangi L. (2019). mRNA-Based Protein Replacement Therapy for the Heart. Mol. Ther..

[65] Goldstein A.L., Slater F.D., White A. (1966). Preparation, assay, and partial purification of a thymic lymphocytopoietic factor (thymosin). Proc. Natl. Acad. Sci. USA.

[66] Huff T., Müller C.S., Otto A.M., Netzker R., Hannappel E. (2001). β-Thymosins, small acidic peptides with multiple functions. Int. J. Biochem. Cell Biol..

[67] Hannappel E. (2007). Beta-Thymosins. Ann. N.Y. Acad. Sci..

[68] Hoch K., Volk D.E., Litwack G. (2016). Chapter One—Structures of Thymosin Proteins. Vitamins and Hormones.

[69] Mora C.A., Baumann C.A., Paino J.E., Goldstein A.L., Badamchian M. (1997). Biodistribution of synthetic thymosin beta 4 in the serum, urine, and major organs of mice. Int. J. Immunopharmacol..

[70] Hannappel E., Xu G.J., Morgan J., Hempstead J., Horecker B.L. (1982). Thymosin beta 4: A ubiquitous peptide in rat and mouse tissues. Proc. Natl. Acad. Sci. USA.

[71] Xu G.J., Hannappel E., Morgan J., Hempstead J., Horecker B.L. (1982). Synthesis of thymosin beta 4 by peritoneal macrophages and adherent spleen cells. Proc. Natl. Acad. Sci. USA.

[72] Hannappel E., Leibold W. (1985). Biosynthesis rates and content of thymosin beta 4 in cell lines. Arch. Biochem. Biophys..

[73] Lin S.C., Morrison-Bogorad M. (1990). Developmental expression of mRNAs encoding thymosins beta 4 and beta 10 in rat brain and other tissues. J. Mol. Neurosci..

[74] Nemolato S., Cabras T., Fanari M., Cau F., Fanni D., Gerosa C., Manconi B., Messana I., Castagnola M., Faa G. (2010). Immunoreactivity of thymosin beta 4 in human foetal and adult genitourinary tract. Eur. J. Histochem..

[75] Salhab M., Papillier P., Perreau C., Guyader-Joly C., Dupont J., Mermillod P., Uzbekova S. (2010). Thymosins β-4 and β-10 are expressed in bovine ovarian follicles and upregulated in cumulus cells during meiotic maturation. Reprod. Fertil. Dev..

[76] Gerosa C., Fanni D., Nemolato S., Locci A., Marinelli V., Cabras T., Messana I., Castagnola M., Monga G., Fanos V. (2010). Thymosin beta-10 expression in developing human kidney. J. Matern. Neonatal Med..

[77] Nemolato S., Cabras T., Cau F., Fanari M.U., Fanni D., Manconi B., Messana I., Castagnola M., Faa G. (2010). Different Thymosin Beta 4 Immunoreactivity in Foetal and Adult Gastrointestinal Tract. PLoS ONE.

[78] Fanni D., Gerosa C., Nemolato S., Locci A., Marinelli V., Cabras T., Messana I., Fanos V., Castagnola M., Faa G. (2011). Thymosin beta 10 expression in developing human salivary glands. Early Hum. Dev..

[79] Rossdeutsch A., Smart N., Dubé K.N., Turner M., Riley P.R. (2012). Essential role for thymosin beta4 in regulating vascular smooth muscle cell development and vessel wall stability. Circ. Res..

[80] Shin S.-H., Lee S., Bae J.-S., Jee J.-G., Cha H.-J., Lee A.Y.M. (2014). Thymosin Beta4 Regulates Cardiac Valve Formation Via Endothelial-Mesenchymal Transformation in Zebrafish Embryos. Mol. Cells.

[81] Saunders V., Dewing J.M., Sanchez-Elsner T., Wilson D.I. (2018). Expression and localisation of thymosin beta-4 in the developing human early fetal heart. PLoS ONE.

[82] Yang H.M., Kang S.W., Sung J., Kim K., Kleinman H. (2020). Purinergic Signaling Involvement in Thymosin beta4-mediated Corneal Epithelial Cell Migration. Curr. Eye Res..

[83] Padmanabhan K., Grobe H., Cohen J., Soffer A., Mahly A., Adir O., Zaidel-Bar R., Luxenburg C. (2020). Thymosin β4 is essential for adherens junction stability and epidermal planar cell polarity. Development.

[84] Mantri M., Scuderi G.J., Abedini-Nassab R., Wang M.F.Z., McKellar D., Shi H., Grodner B., Butcher J.T., De Vlaminck I. (2021). Spatiotemporal single-cell RNA sequencing of developing chicken hearts identifies interplay between cellular differentiation and morphogenesis. Nat. Commun..

[85] Dathe V., Brand-Saberi B. (2004). Expression of thymosin beta4 during chick development. Brain Struct. Funct..

[86] Gómez-Márquez J., del Amo F.F., Carpintero P., Anadón R. (1996). High levels of mouse thymosin beta4 mRNA in differentiating P19 embryonic cells and during development of cardiovascular tissues. Biochim. Biophys. Acta.

[87] Bock-Marquette I., Saxena A., White M.D., DiMaio J.M., Srivastava D. (2004). Thymosin beta4 activates integrin-linked kinase and promotes cardiac cell migration, survival and cardiac repair. Nature.

[88] Hoff M.J.V.D., Moorman A.F., Ruijter J.M., Lamers W.H., Bennington R.W., Markwald R.R., Wessels A. (1999). Myocardialization of the Cardiac Outflow Tract. Dev. Biol..

[89] Smart N., Risebro C.A., Melville A.A.D., Moses K., Schwartz R.J., Chien K.R., Riley P.R. (2007). Thymosin beta-4 Is Essential for Coronary Vessel Development and Promotes Neovascularization via Adult Epicardium. Ann. N.Y. Acad. Sci..

[90] Banerjee I., Morris T.M., Evans S.M., Chen J. (2013). and Chen, J. Thymosin beta4 is not required for embryonic viability or vascular development. Circ. Res..

[91] Smart N., Risebro C.A., Melville A.A., Moses K., Schwartz R.J., Chien K.R., Riley P.R. (2007). Thymosin beta4 induces adult epicardial progenitor mobilization and neovascularization. Nature.

[92] Banerjee I., Zhang J., Moore-Morris T., Lange S., Shen T., Dalton N.D., Gu Y., Peterson K.L., Evans S.M., Chen J. (2012). Thymosin beta 4 is dispensable for murine cardiac development and function. Circ. Res..

[93] Dubé K.N., Smart N. (2018). Thymosin β4 and the vasculature: Multiple roles in development, repair and protection against disease. Expert Opin. Biol. Ther..

[94] Faa G., Piras M., Mancuso L., Coni P., Pichiri G., Orrù G., Fanni D., Gerosa C., Cao G., Taibi R. (2021). Thymosin beta-4 prenatal administration improves fetal development and halts side effects due to preterm delivery. Eur. Rev. Med. Pharmacol. Sci..

[95] Muñoz-Espín D., Serrano M. (2014). Cellular senescence: From physiology to pathology. Nat. Rev. Mol. Cell Biol..

[96] Kirkwood T.B., Austad S.N. (2000). Why do we age?. Nature.

[97] Hinkel R., Ball H.L., DiMaio J.M., Shrivastava S., Thatcher J.E., Singh A.N., Sun X., Faskerti G., Olson E.N., Kupatt C. (2015). C-terminal variable AGES domain of Thymosin beta4: The molecule’s primary contribution in support of post-ischemic cardiac function and repair. J. Mol. Cell. Cardiol..

[98] Young J.D., Lawrence A.J., MacLean A.G., Leung B.P., McInnes I.B., Canas B., Pappin D.J.C., Stevenson R.D. (1999). Thymosin beta 4 sulfoxide is an anti-inflammatory agent generated by monocytes in the presence of glucocorticoids. Nat. Med..

[99] Belsky J.B., Rivers E.P., Filbin M.R., Lee P.J., Morris D.C. (2018). Thymosin beta 4 regulation of actin in sepsis. Expert Opin. Biol. Ther..

[100] Carion T.W., Ebrahim A.S., Kracht D., Agrawal A., Strand E., Kaddurah O., McWhirter C.R., Sosne G., Berger E.A. (2018). Thymosin Beta-4 and Ciprofloxacin Adjunctive Therapy Improves Pseudomonas Aeruginosa-Induced Keratitis. Cells.

[101] Sosne G., Qiu P., Kurpakus-Wheater M. (2007). Thymosin beta 4: A novel corneal wound healing and anti-inflammatory agent. Clin. Ophthalmol..

[102] Ho E.N., Kwok W.H., Lau M.Y., Wong A.S., Wan T.S., Lam K.K., Schiff P.J., Stewart B.D. (2012). Doping control analysis of TB-500, a synthetic version of an active region of thymosin beta(4), in equine urine and plasma by liquid chromatography-mass spectrometry. J. Chromatogr. A.

[103] Kleinman H.K., Sosne G. (2016). Thymosin beta4 Promotes Dermal Healing. Vitam. Horm..

[104] Yang W.S., Kang S., Sung J., Kleinman H.K. (2019). Thymosin beta4: Potential to treat epidermolysis bullosa and other severe dermal injuries. Eur. J. Dermatol..

[105] Hinkel R., Klett K., Bähr A., Kupatt C. (2018). Thymosin beta4-mediated protective effects in the heart. Expert Opin. Biol. Ther..

[106] Srivastava D., Ieda M., Fu J., Qian L. (2012). Cardiac repair with thymosin β4 and cardiac reprogramming factors. Ann. N.Y. Acad. Sci..

[107] Peng H., Xu J., Yang X.P., Dai X., Peterson E.L., Carretero O.A., Rhaleb N.E. (2014). Thymosin-β4 prevents cardiac rupture and improves cardiac function in mice with myocardial infarction. Am. J. Physiol. Circ. Physiol..

[108] Huang Z., Song Y., Pang Z., Zhang B., Yang H., Shi H., Chen J., Gong H., Qian J., Ge J. (2017). Targeted delivery of thymosin beta 4 to the injured myocardium using CREKA-conjugated nanoparticles. Int. J. Nanomed..

[109] Quan Z., Wang Q.-L., Zhou P., Wang G.-D., Tan Y.-Z., Wang H.-J. (2017). Thymosin β4 promotes the survival and angiogenesis of transplanted endothelial progenitor cells in the infarcted myocardium. Int. J. Mol. Med..

[110] Zhao Y., Song J., Bi X., Gao J., Shen Z., Zhu J., Fu G. (2018). Thymosin β4 promotes endothelial progenitor cell angiogenesis via a vascular endothelial growth factor-dependent mechanism. Mol. Med. Rep..

[111] Kassem K.M., Vaid S., Peng H., Sarkar S., Rhaleb N.E. (2019). Tβ4-Ac-SDKP pathway: Any relevance for the cardiovascular system?. Can. J. Physiol. Pharmacol..

[112] Bjørklund G., Dadar M., Aaseth J., Chirumbolo S. (2020). Thymosin β4: A Multi-Faceted Tissue Repair Stimulating Protein in Heart Injury. Curr. Med. Chem..

[113] Wang Y.-L., Yu S.-N., Shen H.-R., Wang H.-J., Wu X.-P., Wang Q.-L., Zhou B., Tan Y.-Z. (2021). Thymosin β4 released from functionalized self-assembling peptide activates epicardium and enhances repair of infarcted myocardium. Theranostics.

[114] Gladka M.M., Kohela A., Molenaar B., Versteeg D., Kooijman L., Monshouwer-Kloots J., Kremer V., Vos H.R., Huibers M.M.H., Haigh J.J. (2021). Cardiomyocytes stimulate angiogenesis after ischemic injury in a ZEB2-dependent manner. Nat. Commun..

[115] Bock-Marquette I., Shrivastava S., Pipes G.T., Thatcher J.E., Blystone A., Shelton J.M., Galindo C.L., Melegh B., Srivastava D., Olson E.N. (2009). Thymosin beta4 mediated PKC activation is essential to initiate the embryonic coronary developmental program and epicardial progenitor cell activation in adult mice in vivo. J. Mol. Cell Cardiol..

[116] Lepilina A., Coon A.N., Kikuchi K., Holdway J.E., Roberts R.W., Burns C.G., Poss K.D. (2006). A dynamic epicardial injury response supports progenitor cell activity during zebrafish heart regeneration. Cell.

[117] Cao Y., Duca S., Cao J. (2019). Epicardium in Heart Development. Cold Spring Harb. Perspect. Biol..

[118] Cao J., Poss K.D. (2018). The epicardium as a hub for heart regeneration. Nat. Rev. Cardiol..

[119] Cao Y., Cao J. (2018). Covering and Re-Covering the Heart: Development and Regeneration of the Epicardium. J. Cardiovasc. Dev. Dis..

[120] Wessels A., Pérez-Pomares J. (2004). The epicardium and epicardially derived cells (EPDCs) as cardiac stem cells. Anat. Rec. A Discov. Mol. Cell. Evol. Biol..

[121] Quijada P., Trembley M.A., Small E.M. (2020). The Role of the Epicardium during Heart Development and Repair. Circ. Res..

[122] Männer J. (1992). The development of pericardial villi in the chick embryo. Brain Struct. Funct..

[123] Lie-Venema H., Akker N.M.S.V.D., Bax N.A.M., Winter E.M., Maas S., Kekarainen T., Hoeben R.C., DeRuiter M.C., Poelmann R.E., Groot A.C.G.-D. (2007). Origin, Fate, and Function of Epicardium-Derived Cells (EPDCs) in Normal and Abnormal Cardiac Development. Sci. World, J..

[124] Katz T.C., Singh M., Degenhardt K., Rivera-Feliciano J., Johnson R.L., Epstein J.A., Tabin C.J. (2012). Distinct Compartments of the Proepicardial Organ Give Rise to Coronary Vascular Endothelial Cells. Dev. Cell.

[125] Cai C.-L., Martin J.C., Sun Y., Cui L., Wang L., Ouyang K., Yang L., Bu L., Liang X., Zhang X. (2008). A myocardial lineage derives from Tbx18 epicardial cells. Nat. Cell Biol..

[126] Zhou B., Ma Q., Rajagopal S., Wu S.M., Domian I., Rivera-Feliciano J., Jiang D., Von Gise A., Ikeda S., Chien K.R. (2008). Epicardial progenitors contribute to the cardiomyocyte lineage in the developing heart. Nat. Cell Biol..

[127] Yamaguchi Y., Cavallero S., Patterson M., Shen H., Xu J., Kumar S.R., Sucov H.M. (2015). Adipogenesis and epicardial adipose tissue: A novel fate of the epicardium induced by mesenchymal transformation and PPARgamma activation. Proc. Natl. Acad. Sci. USA.

[128] Porrello E.R., Olson E.N. (2014). A neonatal blueprint for cardiac regeneration. Stem Cell Res..

[129] Lu J., Richardson J.A., Olson E.N. (1998). Capsulin: A novel bHLH transcription factor expressed in epicardial progenitors and mesenchyme of visceral organs. Mech. Dev..

[130] Kumar N., Liao T.D., Romero C.A., Maheshwari M., Peterson E.L., Carretero O.A. (2018). Thymosin beta4 Deficiency Exacerbates Renal and Cardiac Injury in Angiotensin-II-Induced Hypertension. Hypertension.

[131] Morita T., Hayashi K. (2018). Tumor Progression Is Mediated by Thymosin-beta4 through a TGFbeta/MRTF Signaling Axis. Mol. Cancer Res..

[132] Sribenja S., Wongkham S., Wongkham C., Yao Q., Chen C. (2013). Roles and mechanisms of beta-thymosins in cell migration and cancer metastasis: An update. Cancer Investig..

[133] Wong H.Y., Wang G.M., Croessmann S., Zabransky D.J., Chu D., Garay J.P., Cidado J., Cochran R.L., Beaver J.A., Aggarwal A. (2015). TMSB4Y is a candidate tumor suppressor on the Y chromosome and is deleted in male breast cancer. Oncotarget.

[134] Caers J., Otjacques E., Hose D., Klein B., Vanderkerken K. (2010). Thymosin beta4 in multiple myeloma: Friend or foe. Ann. N.Y. Acad. Sci..

[135] Caers J., Hose D., Kuipers I., Bos T.J., Van Valckenborgh E., Menu E., De Bruyne E., Goldschmidt H., Van Camp B., Klein B. (2010). Thymosin beta4 has tumor suppressive effects and its decreased expression results in poor prognosis and decreased survival in multiple myeloma. Haematologica.

[136] Goldstein A.L., Kleinman H.K. (2015). Advances in the basic and clinical applications of thymosin beta4. Expert Opin. Biol. Ther..

[137] Schneider M.D. (2004). Regenerative medicine: Prometheus unbound. Nat. Cell Biol..

